# The Canadian Association of Gastroenterology’s New Climate Change Committee

**DOI:** 10.1093/jcag/gwae006

**Published:** 2024-02-21

**Authors:** Desmond Leddin, Harminder Singh, David Armstrong, Kelsey Cheyne, Ciaran Galts, John Igoe, Grigorios Leontiadis, Jerry McGrath, Cara Pray, Daniel Sadowski, Neal Shahidi, Paul Sinclair, Frances Tse, Russell Yanofsky

**Affiliations:** Division of Digestive Care & Endoscopy, Department of Medicine, Dalhousie University, Halifax, NS B3H 4R2, Canada; Section of Gastroenterology, Deparment of Internal Medicine, University of Manitoba, Winnipeg, MB R3E 3P4, Canada; Division of Gastroenterology, McMaster University, Hamilton, ON L8S 4K1, Canada; Canadian Digestive Health Foundation, Oakville, ON L6M 4J2, Canada; Division of Gastroenterology, McMaster University, Hamilton, ON L8S 4K1, Canada; Division of Digestive Care & Endoscopy, Department of Medicine, Dalhousie University, Halifax, NS B3H 4R2, Canada; Division of Gastroenterology, McMaster University, Hamilton, ON L8S 4K1, Canada; Department of Medicine, Memorial University, St. John’s, NL A1B 3V6, Canada; Division of Gastroenterology, McMaster University, Hamilton, ON L8S 4K1, Canada; Department of Medicine, University of Alberta, Edmonton, AB T6G 2G3, Canada; Division of Gastroenterology, Department of Medicine, University of British Columbia, Vancouver, BC V5Z 1M9, Canada; INSINC Consulting Inc., Guelph, ON N1G 3G3, Canada; Division of Gastroenterology, McMaster University, Hamilton, ON L8S 4K1, Canada; Division of Gastroenterology, University of Toronto, Toronto, ON M5S 1A1, Canada

The board of the Canadian Association of Gastroenterology has approved the formation of a new committee (members listed below) focussed on the intersection of environmental change and digestive health.

In 2021, a special interest group (SIG) was formed around this topic. The SIG was involved in networking with other peer associations active in this area, giving small group sessions at CDDW, measuring the travel-related emissions of the annual CDDW meeting, and measuring the carbon footprint of endoscopic practice. In 2023, with increasing recognition of the impact of environmental change on digestive health and disease and the fact that the SIG had demonstrated the ability to function, the board decided to establish a formal committee.

Our environment is changing because of 3 interconnected drivers—climate change, pollution, and biodiversity loss.^1^ Environmental change is now recognized as a major public health challenge.^2^

Digestive health and disease are being impacted by environmental change.^3^ For example, atmospheric warming is leading to an increased frequency of extreme weather events which affect the delivery of care, as seen during severe flooding and wildfire events. There is increasing awareness of the role pollution may play in the exacerbation, or incidence, of digestive disease. Although it is early, and the evidence is still accumulating, there are signals that airborne pollution may contribute to the incidence of inflammatory bowel disease^4,5^ and occurrence of some malignancies, including oesophageal tumours.^6^ Biodiversity loss compromises crop and fishery yields, the maintenance of clean water supplies, and prevention of several infectious diseases. Clearly, it is in the interest of the digestive health community to engage on these issues.

An argument can be made that we have professional responsibilities to meet this challenge to public health. Canadian healthcare contributes about 5% of total national emissions.^7^ These emissions of greenhouse gases and pollutants are adversely affecting public health. It is unlikely that we will ever reduce health-related emissions to zero, but it does follow that ethically we have a duty to reduce them as much as possible. There is also an issue of intergenerational justice. It is not fair or just for us to live beyond our means and pass the problem on generations yet to come. We have professional duties of advocacy—especially important since we know that environmental change will disproportionately impact the most vulnerable sections of society, including low income, children, and the elderly.

Few remain convinced we should not engage. Some argue that nothing can be done. That clearly is not true as evidenced by the National Health Service in the United Kingdom, which has significantly cut health-related emissions.^8^ Others argue that any reduction or contribution that we make would be so small on the global scale as to be insignificant. There is truth in that, but the process of change must begin somewhere. When countries as wealthy as Canada decline to engage on this issue it sends a signal globally to others who are less well-off that they should not bother. It becomes a self-perpetuating circle of inaction. Some may feel that it is not the problem of the healthcare sector, but it is up to governments to sort this. The unfortunate reality is that governments are unable to solve this on their own. While there are some signs of progress, the government is in a very difficult position, and it needs our expertise, support, and activism to make the necessary change away from fossil fuels.

Regarding what we, CAG can do, the priority is probably to educate ourselves, so that all digestive health providers are more aware of the problem and can move to action. We, the gastroenterologists, can make changes, and even if they are small, they may lead to system-wide changes when we engage with our nursing colleagues and hospital managers. As a start, we can ensure that we make the best, most efficient use of the resources that are available to us. The most expensive, inefficient activities are those that are unnecessary; practice optimization to ensure that minimize unnecessary, ineffective investigations and therapies will reduce our carbon footprint, free up resources for those in need, and reduce potential harms. Adoption of telehealth strategies, particularly in a country as large as Canada, can reduce the indirect costs and carbon footprint of health care delivery and, in many cases, decrease patient burden.

Sadly, the European Union’s Copernicus Climate Change Service has just announced that 2023 “was the planet’s hottest on record by a substantial margin and likely the world’s warmest in the last 100,000 years”.^9^ However, changes are underway. Emissions are falling in many countries worldwide with renewable energy now comparable in price to fossil fuels and the recent Congress of the Parties to the Paris agreement committed to move away from fossil fuels. There is a lot that we can do to add to this momentum. Within the global digestive health community many societies have established climate committees, sustainability plans are being developed and a comprehensive course on the topic has been published.^10^ The issue of environmental change is one that will define our future and that of generations to come. The Climate Committee of the CAG, with the help and support of the membership, is committed to making a difference.

Desmond Leddin Co-Chair Climate Committee, CAG

Harminder Singh Co-Chair Climate Committee, CAG

Jerry McGrath VP Administrative Affairs, CAG

***CAG Climate Committee Members***

David Armstrong, Ciaran Galts PGY5, John Igoe

Grigorios Leontiadis, Cara Pray PGY6, Dan Sadowski

Paul Sinclair, Neal Shahidi, Frances Tse

Russell Yanofsky PGY5

***Representative for the Canadian Digestive Health Foundation***

Kelsey Cheyne

## Supplementary Material

gwae006_suppl_Supplementary_Materials

## Data Availability

No new data were generated or analysed in support of this editorial.
