# Supplementary material for: The Canadian Association of Gastroenterology’s New Climate Change Committee
Source: J Can Assoc Gastroenterol. 2024 Feb 21;7(2):135–6. doi: 10.1093/jcag/gwae006 (PMC10999766; doi:10.1093/jcag/gwae006)
Supplement: gwae006_suppl_Supplementary_Materials [file gwae006_suppl_supplementary_materials.zip › ICMJE_Armstrong.docx]

| ICMJE DISCLOSURE FORM | |
| --- | --- |
| **Date:** | 1/21/2024 |
| **Your Name:** | David Armstrong |
| **Manuscript Title:** | The Canadian Association of Gastroenterology Climate Committee |
| **Manuscript Number (if known):** | Click or tap here to enter text. |
| In the interest of transparency, we ask you to disclose all relationships/activities/interests listed below that are related to the content of your manuscript. “Related” means any relation with for-profit or not-for-profit third parties whose interests may be affected by the content of the manuscript. Disclosure represents a commitment to transparency and does not necessarily indicate a bias. If you are in doubt about whether to list a relationship/activity/interest, it is preferable that you do so.  The author’s relationships/activities/interests should be defined broadly. For example, if your manuscript pertains to the epidemiology of hypertension, you should declare all relationships with manufacturers of antihypertensive medication, even if that medication is not mentioned in the manuscript.  In item #1 below, report all support for the work reported in this manuscript without time limit. For all other items, the time frame for disclosure is the past 36 months. | |

|  | | | **Name all entities with whom you have this relationship or indicate none (add rows as needed)** | **Specifications/Comments (e.g., if payments were made to you or to your institution)** |
| --- | --- | --- | --- | --- |
| **Time frame: Since the initial planning of the work** | | | | |
| **1** | All support for the present manuscript (e.g., funding, provision of study materials, medical writing, article processing charges, etc.)  **No time limit for this item.** | | \|  \| **None** \| \| --- \| --- \|  \|  \|  \| \| --- \| --- \| \|  \|  \| \|  \| Click the tab key to add additional rows. \| | |
| **Time frame: past 36 months** | | | | |
| **2** | | Grants or contracts from any entity (if not indicated in item #1 above). | \|  \| **None** \| \| --- \| --- \|  \| Nestlé Health Sciences \| Research; payment to institution \| \| --- \| --- \| \| Weston Family Foundation \| Research; payment to institution \| \|  \|  \| | |
| **3** | | Royalties or licenses | \|  \| **None** \| \| --- \| --- \|  \|  \|  \| \| --- \| --- \| \|  \|  \| \|  \|  \| | |
| **4** | | Consulting fees | \|  \| **None** \| \| --- \| --- \|  \| Canadian Partnership Against Cancer \| Expert Advisor; Stipend to me \| \| --- \| --- \| \| The Scripps Research Institute / CALIBR \| Honorarium to me \| \|  \|  \| \|  \|  \| | |
| **5** | | Payment or honoraria for lectures, presentations, speakers bureaus, manuscript writing or educational events | \|  \| **None** \| \| --- \| --- \|  \| Viatris \| Payment to me for lecture \| \| --- \| --- \| \| Takeda \| Payment to me for lecture \| \| Fresenius Kabi \| Payment to me for lecture \| | |
| **6** | | Payment for expert testimony | \|  \| **None** \| \| --- \| --- \|  \| McCarthy Tetrault \| Payment to me \| \| --- \| --- \| \| Dives, Harper, Stanger & Mizrahi \| Payment to me \| \|  \|  \| | |
| **7** | | Support for attending meetings and/or travel | \|  \| **None** \| \| --- \| --- \|  \| Canadian Partnership Against Cancer (CPAC) \| Travel and accommodation for CPAC meetings \| \| --- \| --- \| \| European Commission Initiative on Colorectal  Cancer \| Travel & Accommodation for ECICC Guideline  Methodology Meeting (Summer 2019) \| \| International Working Group for the Classification of Oesophagitis \| Travel and accommodation for Consensus Meeting participation \| | |
| **8** | | Patents planned, issued or pending | \|  \| **None** \| \| --- \| --- \|  \| A.I. VALI Inc. \| No payments to me or the institution \| \| --- \| --- \| \|  \|  \| \|  \|  \| | |
| **9** | | Participation on a Data Safety Monitoring Board or Advisory Board | \|  \| **None** \| \| --- \| --- \|  \| Sanofi \| Advisory Board, Honorarium to me \| \| --- \| --- \| \| Cinclus Pharma \| Advisory Board, No payment \| \| Phathom Pharma \| Advisory Board, No payment \| \| Takeda Canada \| Advisory Board, Honorarium to me \| | |
| **10** | | Leadership or fiduciary role in other board, society, committee or advocacy group, paid or unpaid | \|  \| **None** \| \| --- \| --- \|  \| Canadian Digestive Health Foundation (CDHF) \| Board Member; No payment \| \| --- \| --- \| \| Int. Working Group Classification Oesophagitis \| Board Member; Treasurer with stipend to me \| \| A.I. VALI Inc. \| Co-Founder; No payment \| | |
| **11** | | Stock or stock options | \|  \| **None** \| \| --- \| --- \|  \|  \|  \| \| --- \| --- \| \|  \|  \| \|  \|  \| | |
| **12** | | Receipt of equipment, materials, drugs, medical writing, gifts or other services | \|  \| **None** \| \| --- \| --- \|  \|  \|  \| \| --- \| --- \| \|  \|  \| \|  \|  \| | |
| **13** | | Other financial or non-financial interests | \|  \| **None** \| \| --- \| --- \|  \|  \|  \| \| --- \| --- \| \|  \|  \| \|  \|  \| | |
|  | |  |  | |
| **Please place an “X” next to the following statement to indicate your agreement:** | | | | |
|  | | I certify that I have answered every question and have not altered the wording of any of the questions on this form. | | |
